# Texture Analysis of T2-Weighted Images as Reliable Biomarker of Chronic Kidney Disease Microstructural State

**DOI:** 10.3390/biomedicines13061381

**Published:** 2025-06-04

**Authors:** Marcin Majos, Artur Klepaczko, Katarzyna Szychowska, Ludomir Stefanczyk, Ilona Kurnatowska

**Affiliations:** 1 Department of Normal and Clinical Anatomy, Medical University of Lodz, 90-419 Lodz, Poland; 2 I Department of Radiology and Diagnostic Imaging, Medical University of Lodz, 90-419 Lodz, Poland; 3 Medical Electronics Division, Institute of Electronics, Lodz University of Technology, 90-924 Lodz, Poland; 4 Department of Internal Diseases and Transplant Nephrology, Medical University of Lodz, 90-419 Lodz, Poland

**Keywords:** chronic kidney disease, CKD, magnetic resonance, MRI, texture analysis, T2-weighted images

## Abstract

**Objectives**: The diagnostics of chronic kidney disease (CKD) consist of three basic groups of examinations: laboratory tests, radiological imaging and histopathological examinations. However, in the most severe clinical cases, where a fast, undisputed decision is required, histopathological tests are the only suitable option. Unfortunately, such tests require an invasive kidney biopsy, which is not possible in many patients. The aim of this study is to create an algorithm that can categorize CKD patients into active and non-active phases on the basis of MRI texture analysis and compare the results with histopathological examinations. **Methods**: MRI examinations were performed on healthy volunteers (group 1, N = 14) and CKD patients who also received kidney biopsy. The histopathological examination was used to divide the patients into active phase CKD (group 2, N = 58) and non-active phase CKD (group 3, N = 22). The T2-weighted MRI images were analyzed using a Support Vector Machine (SVM) model created with qMazDa software, which was trained to classify images into the appropriate group of CKD activity. **Results**: The following evaluation metrics were calculated for the final SVM models corresponding to confusion matrices: for texture analysis—balanced accuracy 81.6%, sensitivity 68.2–92.0%, specificity 82.5–97.5% and precision 62.5–95.8%; for texture and shape analysis—balanced accuracy 87.3%, sensitivity 77.3–100.0%, specificity 87.5–100.0% and precision 65.4–100.0%. **Conclusions**: Texture analysis of T2-weighted images associated with kidney shape features seems to be reliable method of assessing the state of ongoing CKD.

## 1. Introduction

Chronic kidney disease (CKD) represents an extended period of deterioration in kidney function [1]. In many cases, CKD arises from a single kidney injury that did not respond to standard healing processes and which was exacerbated over time by the persistence of a pathological factor. Nowadays, the most common causes of CKD are hypertension and diabetes [2,3]; in both cases, kidney microstructure is altered by a self-reinforcing mechanism beginning with hyperfiltration via arteriolar remodeling, followed by increasing levels of glycation end products, various cytokines and growth factors [4,5,6,7,8,9]. Hyperfiltration leads to thickening of the basement membrane, degeneration of podocytes and glomerular remodeling. Eventually, the kidney demonstrates proteinuria and activation of inflammatory cascades, leading to a final loss of function by glomerular sclerosis, renal fibrosis and scarring. While this process is constant, the intensity varies, proceeding via a series of active phases and non-active ones. In clinical management, is crucial to distinguish the onset of the active phase of CKD, as it demands fast and intensive pharmacological treatment.

CKD diagnostics comprises three basic groups of examinations: laboratory tests, radiological imaging and histopathological examinations [10]. In clinical practice, the most widespread and most accessible means of kidney diagnostics is laboratory testing, which provides a reliable and up-to-date insight into renal function [11]. Radiological examinations, typically ultrasound, are able to present morphological changes such as shape, size or cortico-medullary differentiation, which are useful for standard monitoring of CKD [12]. However, in the most severe cases, when a fast and undisputed clinical decision is required, the only approach capable of fulfilling this need is histopathological testing. Unfortunately, performing histopathological tests requires the collection of kidney biopsy, an invasive procedure burdened with sometimes severe complications and various contraindications which can exclude many patients. Fortunately, recent advances in diagnostic imaging, particularly magnetic resonance imaging (MRI), offer hope to patients disqualified for invasive procedures; this novel approach to image analysis seems to be a promising alternative to kidney biopsy in cases the latter is burdened by excessive risk to the patient [13,14,15].

The aim of this study is to create an algorithm which will be able to divide CKD patients into those with active and non-active phase disease on the basis of MRI texture analysis and compare the findings with histopathological examinations.

## 2. Materials and Methods

Two groups of patients were included in this study. The first (Group 1) consisted of healthy volunteers without any known kidney pathology and who had received an MRI examination in the Radiology Department of our hospital. The second included consecutive patients suffering from CKD admitted to Department of Nephrology of our hospital; all had received a right kidney biopsy and control MRI examination after 24 h. The histopathological outcome was used to divide the patients into two subgroups: those with the active phase of CKD (Group 2) and those with the non-active phase of CKD (Group 3). After excluding examinations with poor SNR, 14 people were included in Group 1, 58 in Group 2 and 22 in Group 3 (Table 1). The patients of Group 2 and Group 3 did not receive any additional pharmacological treatment prior to biopsy and MRI examination.

The patients enrolled in the trial suffered from the following: focal segmental glomerular sclerosis (Group 2—eleven patients; Group 3—seven patients), vasculitis (Group 2—eleven patients; Group 3—one patient), lupus nephritis (Group 2—seven patients; Group 3—no patients), tubulointerstitial nephritis (Group 2—eight patients; Group 3—one patient), tubular nephropathy (Group 2—no patients; Group 3—one patient), IgA nephropathy (Group 2—nine patients; Group 3—two patients), membranous nephropathy (Group 2—twelve patients; Group 3—no patients) and diabetes-related nephropathy (Group 2—no patients; Group 3—three patients). In addition, seven patients had end-stage kidney disease.

All patients received an MRI scan using a 3T Magnetom Vida (Siemens Healthcare GmbH, Erlangen, Germany) a day before kidney biopsy or the day after. For the patients scanned before biopsy, both kidneys were included in the further analysis; for those examined after biopsy, only one kidney was included. The MRI examination protocol included T2-weighted HASTE images with fat saturation (TR = 1350 ms, TE = 80 ms, TA = 0.51 ms).

Among Group 1, there were 11 volunteers. All other subjects were patients routinely qualified for diagnosis. Patients suffering from CKD, according to the approved protocol, underwent biopsy on the left kidney before MR examination. Hence, to exclude the impact of biopsy on the subsequent analyses, only the images of the right kidney were processed. This restriction does not apply to Group 1 data, which was free of artefacts caused by renal tissue sample removal. In order to decrease the class imbalance, both left and right kidney from volunteer acquisitions were analyzed, resulting in 25 images of the normal category available for experimentation.

The plan for this prospective trial was accepted by the Bioethical Committee associated with the Medical University of Lodz (decision RNN/206/20/KE, dated 8 September 2020).

### 2.1. Texture Analysis

All feature extraction methods used in the present study are summarized in Table 2. They can be divided into two subgroups. The first group of methods focuses on image texture, whereas the second one concerns the kidney shape. The goal of texture analysis is to extract image features which indicate characteristic intensity distribution patterns. A number of techniques have been devised to statistically model first- and higher-order relationships between image pixels. The present study used statistical descriptors based on histograms, local binary patterns (LBP), gray-level co-occurrence matrix (GLCM) and gray-level run-length matrix (GLRM); in addition, two other feature subsets were derived from autoregressive model (AR) and Haar wavelet (HW) transformation.

All computations were performed in open-source qMaZda software [16], specifically dedicated to image texture analysis. In total, 339 texture features were determined, and the definitions of specific TA parameters are presented in more detail elsewhere [17,18]. qMaZda is equipped with a graphical user interface used to manage various processing tasks, such as delineation of regions of interest within the image, selection of image cross-sections to be processed, determination of required feature extraction methods, image normalization and discretization.

**Figure 1:**
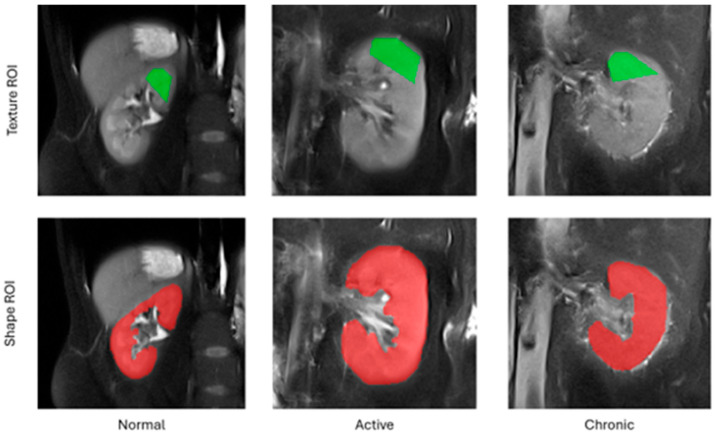
Example regions of interest for texture and shape analysis.

The employed feature extraction workflow comprised the following steps. For each subject included in this study, a middle cross-section of the kidney along the anterior–posterior acquisition axis was selected from the entire 3D volume. Following this, a pentagonal region of interest (ROI) was manually drawn over the tissue area directly above the kidney pelvis (Figure 1). This region was selected for analysis because it is anatomically most representative of the kidney and most repetitively observed on MRI images. A pentagonal ROI was chosen because it was the simplest yet sufficiently flexible shape that could be adjusted to the geometry of a kidney. Next, the pixel intensities within the ROI were normalized to the range defined by the mean intensity of the ROI and the interval of ±3 standard deviations (so-called 3-sigma normalization). The normalization ensures that the computed features are independent from any acquisition settings, scanner parameters or signal energies which may differ between readings, i.e., all factors which affect final image brightness and contrast. Finally, the texture analysis was performed within each ROI, and the computed feature vectors completed with a category label (1—normal, 2—active, 3—chronic) were stored in an output CSV file.

In addition to texture analysis, a series of geometrical attributes were identified based on the entire selected 2D cross-section of the kidney chosen for texture analysis (TA). The set of morphological parameters consisted of 97 features which described kidney shape, such as area, circularity, convexity, principal orientation, contour–skeleton attributes, second-order moments of inertia, or perimeter profile attributes. This group of descriptors was included in the analysis for two reasons: texture alone does not determine all the differences in tissue morphology regarding lesion category, and it is the kidney shape which improves the performance of the ultimate classification model. Each kidney image was represented in the final data set by only one feature vector; as such, there was no risk of data leakage while partitioning the vectors into train and test folds during the exploratory analysis.

### 2.2. Exploratory Analysis

As a relatively large number of textural and geometrical features were extracted for analysis, it was necessary to develop a complex classification rule in a very-high-dimensional space. Such complexity makes a classifier prone to overfitting. Therefore, before proceeding to the training stage, the data must undergo dimensionality reduction, either through feature selection or transformation. The former approach was used in the present study, as it was aimed at identifying the specific texture model and geometrical parameters capable of distinguishing renal tissue states. To this end, a wrapper strategy was employed. Its two main components were the feature space exploration algorithm, which constructs consecutive feature subsets candidates, and a classifier employed to evaluate the discriminative power of those candidates.

Feature space exploration was accomplished using the linear floating forward selection method [19], as implemented in Weka [20] software. Essentially, this search strategy adds subsequent features to a currently selected subset based on their impact on the final classification accuracy, defined as the fraction of all true positive detections out of the total number of data vectors. It must be noted that this simple accuracy metric provides reliable classifier evaluation only in case of uniform class distribution. However, due to the limitation of Weka, this evaluation measure cannot be changed, e.g., to balanced accuracy, which in principle should be used in case of uneven class sizes. Therefore, during the feature selection stage, the dataset was subsampled to contain equal numbers of feature vectors in each category (i.e., 20 samples per class).

At each step of feature space exploration, the obtained accuracy is recorded for future comparisons. Then, the algorithm can optionally take steps backward provided that after adding new features, the removal of some previously selected ones increases the evaluation score at a lower dimensionality. The algorithm terminates when the score does not improve after a predefined number of forward selection steps *d*, which was set to 10 in our experiments. Note, that is this setting the final data dimensionality *n* results from the optimal classifier performance and cannot be assumed a priori.

As regards the classification algorithm, Support Vector Machine (SVM) was selected for both feature evaluation and as a final classifier model. SVM has been found to be robust against problems of class-imbalance, overfitting and outliers, and it demonstrates an inherent capability to model non-linear separating hypersurfaces while maintaining clean, linearized formulation [21]. The latter is obtained by substituting the dot product used in the linear setting with a non-linear kernel function. It implicitly transforms the data into an unknown space of higher dimension, where the class separating boundary becomes linear. One of the popular kernels, used in the present study, is the radial basis function, defined as

$$k\left(x_{i},x_{j}\right)=\exp{\left(-\gamma \left\|x_{i}-x_{j}\right\|\right)}^{2},$$

where *x_i_* and *x_j_* denote data vectors, ||.|| is the *l*^2^-norm and *y* is the kernel parameter which controls the rate of non-linearity introduced to the transformation. Another SVM parameter, usually designated with the letter *C*, relaxes the constraints imposed on the optimization criterion to allow a certain number of training points to violate the decision boundary. This mechanism ensures better model generalization to new data. A common heuristic used to set the value of the *y* parameter is to adjust it to the data dimensionality, i.e., *y* = 1/*n*. This approach was adopted during the feature selection and final classifier training stages, with *C* set to 1. The SVM was evaluated in 5-fold cross-validation mode to enable objective verification of a given feature subset saliency.

After feature selection, the final classifier model was trained using all available data. Also, in this stage, the final classifier was tested using the 5-fold cross-validation method. In order to ensure that features with various value ranges have equal impact on the data similarity metric, all attributes where standardized, so that their mean = 0 and standard deviation = 1. Data standardization was executed both before feature selection and classifier training. Finally, instead of a simple accuracy metric, the classification performance was evaluated using the balanced variant of the measure [22]; in addition, the class-wise true positive rate (TPR, sensitivity), true negative rate (TNR, specificity) and precision were reported. To calculate these measures, all members of a given class were assumed as ‘positive’ examples and the remainder as ‘negative’ ones.

## 3. Results

**Figure 2:**
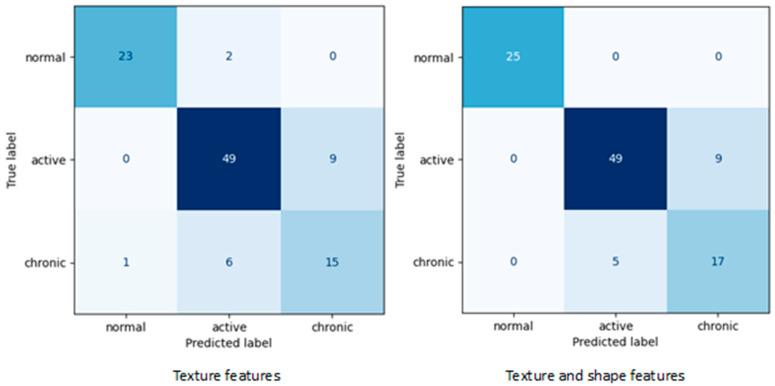
Confusion matrices obtained for final SVM models (Group 1—normal, Group 2—active, Group 3—chronic)—5-fold cross-validation test.

As a result of the first stage of the experiment, various subsets of features relevant to renal tissue classification were identified. Table 3 shows the lists of selected features depending on whether the kidney shape attributes were included in the analysis or not. For the meaning of specific feature names, please refer to the qMazDa manual [18]. Moreover, Table 4 includes all evaluation metrics calculated for final SVM models corresponding to the confusion matrices shown in Figure 2.

**Figure 3:**
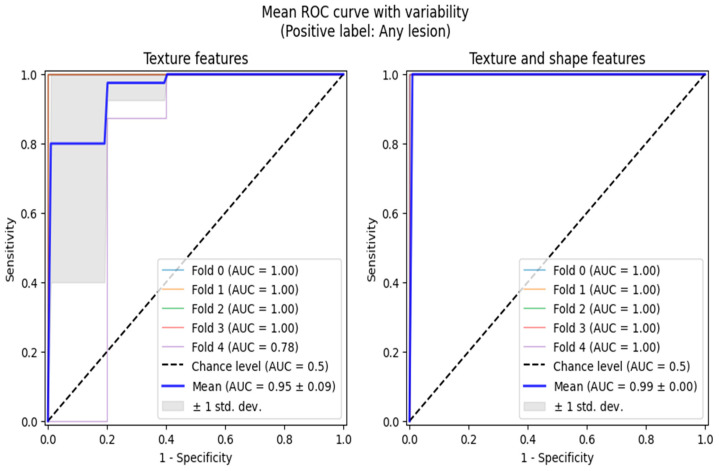
ROC curve analysis between healthy volunteers (Group 1) and patients with CKD (Group 2 combined with Group 3).

**Figure 4:**
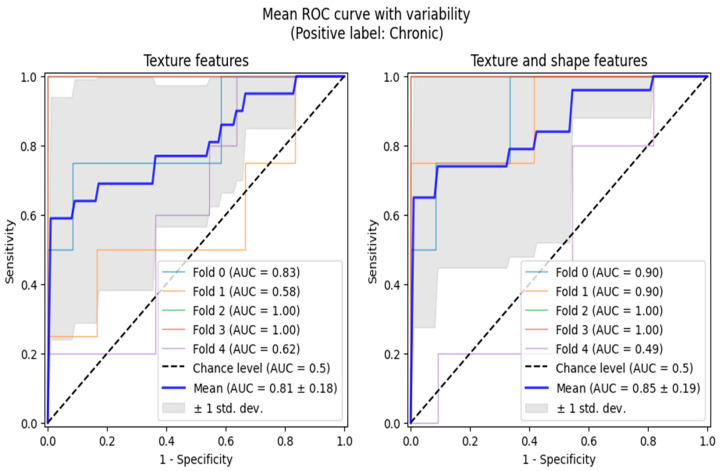
ROC curve analysis between patients with active phase of CKD (Group 2) and patients with non-active phase of CKD (Group 3).

Additionally, we performed ROC analyses in two scenarios, both of which involved reducing the multi-class classification problem to binary ones, adequate for ROC curve analytics. Firstly, we grouped active and chronic subjects into one category and performed normal vs. any lesion analysis. The second analysis was performed only between the chronic and active classes. The ROC analyses were also conducted in the 5-fold cross-validation setting, allowing us to highlight the regions of one standard deviation width around the mean ROC curves (Figure 3 and Figure 4).

## 4. Discussion

Available reports suggest that texture analysis T2-weighted images can be used to effectively assess kidney function. However, the approach presented in this article is intended to have more clinical value, as is can be used to making clinically significant decisions. After all, in everyday medical practice, it is much easier to assess kidney function by taking a blood sample for laboratory tests than performing an MRI scan. On the other hand, MRI is significantly less invasive than performing a diagnostic kidney biopsy.

Modern medicine seeks to minimize the frequency and extent of invasive procedures in both diagnostics and treatment. However, certain invasive procedures, such as biopsies, remain irreplaceable as they offer greater accuracy than laboratory tests or imaging studies. This is a particularly difficult issue with regard to kidney biopsies, as obtaining a histopathological sample may result in dysfunction of the entire organ, often due to vascular complications.

Research into the value of T2-weighted images for the diagnosis of kidney diseases began with animal models. A study on rabbit models by Yusa et al. [23] created a protocol to assess changes in signal intensity during renal artery occlusion; the findings identified the impact of organ hypoperfusion on the prolongation of T2-weighted times. Following this, significant alterations in T2 signal intensities in the kidneys were also reported in an experiment involving 16 rat kidneys with a 9.4T MRI system by Pohlmann et al. [24], in mouse models by Hueper et al. [25] and in transplanted mouse kidneys by Schmidbauer et al. [26]. A particularly noteworthy study by Schley et al. [27] confirmed that T2-weighted signal intensities were related to histopathological features associated with chronic kidney disease (CKD) in mouse models, suggesting that T2 times possess greater diagnostic significance in CKD than T1 times. While these results were derived from mouse models, they are in line with our present conclusions.

Despite positive results from animal models, studies evaluating T2-weighted imaging in human subjects remain rare. Notably, a study of over 140 patients by Inoue et al. [28] identified a correlation between renal fibrosis, hypoxia in the kidneys and the intensity of the T2 signal and ADC maps. Most importantly, the study identified a correlation between MR imaging data and histopathological findings, which was also confirmed in the present study. The literature contains only three reports addressing the role of T2-weighted image textures in diagnosing CKD; however, none of these studies included histopathological examinations of the kidneys, relying solely on GFR results.

Zhang Hao et al. [29] conducted a study involving 55 subjects divided into two groups: healthy volunteers and patients with stage III CKD associated with type II diabetes. All participants underwent MRI examination, which included a T2-weighted sequence and Apparent Diffusion Coefficient (ADC) maps. The algorithm developed by the research team effectively distinguished the healthy subjects from the CKD patients, achieving accuracy values nearing 90%, based on three first-order features and eight texture features derived from the T2-weighted images and the ADC maps. While this study addresses a slightly different facet of CKD progression, focusing more on a decline in kidney function, it confirms the hypothesis that microstructural changes responsible for kidney damage are reflected in the texture of kidney imaging.

Another study on kidney function examined the textures concerning GFR levels in a limited cohort of nine patients following kidney transplantation [30]. While its findings support the correlation between textures and kidney function, caution is warranted due to the small sample size and the use of non-dedicated imaging protocols.

The most recent study on MRI texture analysis of the kidneys was conducted by Yuki Hara et al. [31] Similarly to the present study, the authors evaluated the ability of texture analysis algorithms applied to T1-Dixon and T2*-weighted images, as well as ADC maps, to differentiate between healthy individuals and patients with moderate and severe CKD. The highest discriminative ability was observed for combinatory algorithms, although our algorithm appears to be more effective. This may be related not so much to the design of the algorithm as to the method of patient group classification. Our classification is based on histopathological assessment of existing microstructural changes in the kidneys; in contrast, those participating in the study of Yuki Hara et al. [31] showed clinical symptoms of kidney failure, of various stages, but might not have developed sufficiently advanced alterations in the renal matrix.

Our study has two main limitations. First, the groups included a moderate number of patients, and secondly, the numbers of participants in the two groups were not very well balanced. As such, our findings should be confirmed by further studies with larger groups of patients.

## 5. Conclusions

Texture analysis of T2-weighted images associated with kidney shape features seems to be a reliable method for assessing the state of ongoing CKD.

## Figures and Tables

**Table 1:** Basic demographic characteristics of the participants: Group 1—healthy volunteers; Group 2—patients with active phase of CKD; Group 3—patients with non-active phase of CKD.

Group	N	Gender		Mean Age (Years)	Mean eGFR	Mean Proteinuria (g/24 h)	Number of Patients Treated with Interventional Dialysis Prior to Biopsy
		Female	Male				
1	14	7	7	41.42	>60	---	0
2	58	31	27	51.68	49.7	4.42	8
3	22	10	12	57.99	36.7	2.80	9

**Table 2:** Summary of feature extraction methods.

Texture Model	Model Parameters	Number of Features
Histogram	-	14
LBP	Type: center-symmetric Neighborhood size: 4	4
GLCM	Distances: 1–5 Directions: 0°, 45°, 90°, 135° Type: Symmetrical	280
GLRM	Directions: 0°, 45°, 90°, 135°	28
AR	-	5
HW	Scales: 1, 2 Bands: HH, LH, HL, LL	8
Shape	-	97
	Total:	436

**Table 3:** Selected features with regard to involvement of kidney shape characteristics.

Extraction Method	Selected Features		Total Number of Subset Evaluations	Accuracy [%]
Texture analysis	Gray-level co-occurrence matrix	H2SumEntrp H2ClustPrm H4ClustPrm Z5SumVarnc Z5SumEntrp N1SumOfSqs N4SumVarnc	10,939	81.8
	Local binary patterns	Cs4n2 Cs4n3		
Texture & shape analysis	Histogram	Kurtosis	4281	86.7
	Gray-level run-length matrix	VMGLevNonUn NShrtREmp NMRLNonUni		
	Gray-level co-occurrence matrix	Z1SumAverg Z2SumEntrp Z3SumEntrp Z5SumEntrp N1SumOfSqs N4SumEntrp N5SumVarnc		
	Local binary patterns	Cs4n0 Cs4n2		
	Shape	MzLsz MzW6 CvProfileArea		

**Table 4:** Evaluation metrics for final Support Vector Machine model.

Extraction Method	Balanced Accuracy	Matthews Correlation Coefficient	Class	Sensitivity (TPR)	Specificity (TNR)	Precision	**F1-Score**
Texture	81.6	71.4	Normal	92.0	97.5	95.8	93.9
			Active	84.5	82.5	86.0	85.2
			Chronic	68.2	92.5	62.5	68.2
Texture & Shape	87.3	78.2	Normal	100.0	100.0	100.0	100.0
			Active	84.5	87.5	90.7	87.5
			Chronic	77.3	92.5	65.4	70.8

## Data Availability

The source code is available upon request to interested researchers from the corresponding author.
